# Gut Bacterial Diversity in Different Life Cycle Stages of *Adelphocoris suturalis* (Hemiptera: Miridae)

**DOI:** 10.3389/fmicb.2021.670383

**Published:** 2021-06-02

**Authors:** Hui Xue, Xiangzhen Zhu, Li Wang, Kaixin Zhang, Dongyang Li, Jichao Ji, Lin Niu, Changcai Wu, Xueke Gao, Junyu Luo, Jinjie Cui

**Affiliations:** ^1^State Key Laboratory of Cotton Biology, Institute of Cotton Research, Chinese Academy of Agricultural Sciences, Anyang, China; ^2^Zhengzhou Research Base, State Key Laboratory of Cotton Biology, Zhengzhou University, Zhengzhou, China

**Keywords:** 16S rRNA, microbial composition, symbiotic bacteria, pest control, life stages

## Abstract

Bacteria and insects have a mutually beneficial symbiotic relationship. Bacteria participate in several physiological processes such as reproduction, metabolism, and detoxification of the host. *Adelphocoris suturalis* is considered a pest by the agricultural industry and is now a major pest in cotton, posing a serious threat to agricultural production. As with many insects, various microbes live inside *A. suturalis*. However, the microbial composition and diversity of its life cycle have not been well-studied. To identify the species and community structure of symbiotic bacteria in *A. suturalis*, we used the HiSeq platform to perform high-throughput sequencing of the V3–V4 region in the 16S rRNA of symbiotic bacteria found in *A. suturalis* throughout its life stages. Our results demonstrated that younger nymphs (1st and 2nd instar nymphs) have higher species richness. Proteobacteria (87.06%) and Firmicutes (9.43%) were the dominant phyla of *A. suturalis*. At the genus level, *Erwinia* (28.98%), *Staphylococcus* (5.69%), and *Acinetobacter* (4.54%) were the dominant bacteria. We found that the relative abundance of *Erwinia* was very stable during the whole developmental stage. On the contrary, the relative abundance of *Staphylococcus*, *Acinetobacter*, *Pseudomonas*, and *Corynebacterium* showed significant dynamic changes at different developmental stages. Functional prediction of symbiotic bacteria mainly focuses on metabolic pathways. Our findings document symbiotic bacteria across the life cycle of *A. suturalis*, as well as differences in both the composition and richness in nymph and adult symbiotic bacteria. Our analysis of the bacteria in *A. suturalis* provides important information for the development of novel biological control strategies.

## Introduction

Insects are found almost everywhere in the world. Most insects carry symbiotic microorganisms that are involved in the life cycle processes of the host (Yong et al., 2017; Zhao et al., 2019). There is an interactive relationship between insects and symbiotic bacteria, which play an important role in the health, survival, and behavior of the host (Dillon and Dillon, 2004; Kikuchi et al., 2007; Moran et al., 2008; Santos-Garcia et al., 2017). However, the vast majority of symbiotic microorganisms are concentrated in the intestines of insects, where they act as key regulators of the insect host’s multiple lifestyles (including diet and ecological niche; Gupta and Nair, 2020). Studies have shown that symbiotic bacteria in the intestinal tract of insects can promote host food consumption and digestion; provide immunity and protection against various predators, pathogens, and parasites; control the success rate of host mating and reproduction; provide essential amino acids, metabolic compounds, and nutrients (Russell et al., 2014; Douglas, 2015; Arbuthnott et al., 2016; Wielkopolan and Obrępalska-Stęplowska, 2016; Engl and Kaltenpoth, 2018; Grenier and Leulier, 2020; Horak et al., 2020). In a recent study, the main roles of insect gut bacteria were demonstrated to be providing essential nutrients, followed by digestion and detoxification (Jing et al., 2020). Therefore, insects are highly dependent on intestinal symbiotic bacteria to complete their own growth and development. In short, a very complex and interesting relationship is formed between intestinal symbiotic bacteria and their hosts.

Hemiptera insects have piercing and sucking mouthparts, which not only can directly suck plant juice and kill crops, but also cause serious economic losses by spreading plant viruses. They are notorious agricultural pests (Wang et al., 2015; Liu et al., 2018; Qin et al., 2018). *Adelphocoris suturalis* was originally a minor pest of cotton, but the widespread application of *Bacillus thuringiensis* (Bt) cotton and the reduction of broad-spectrum insecticides has made it a major problem in cotton-growing areas in China (Wu et al., 2008; Lu and Wu, 2011; Luo et al., 2017a). Bt plants can effectively control Lepidopteran pests and can significantly reduce the use of chemical pesticides that are often required in conventional planting systems (Wu et al., 2008; Wang et al., 2018). A major challenge in planting Bt crops to control pests is that insects may evolve resistance to Bt (Wu et al., 2008). *Adelphocoris suturalis* is a typical omnivorous insect, which regularly feeds on crop plants (e.g., cotton and garden pea), other insects like aphids, and occasionally its weaker siblings (Lu and Wu, 2011; Luo et al., 2021). Additionally, *A. suturalis* also has the characteristic of high liquidity (Wu et al., 2010), resulting in poor chemical controls. At present, chemical pesticides such as organophosphates and pyrethroids are widely used in China to control these insects (Zhen et al., 2016). The question of whether these insects are resistant or not needs to be resolved urgently. There is increasing evidence that there is a link between symbiotic bacteria in the insect gut and the evolution of drug resistance (Broderick et al., 2006; Kikuchi et al., 2012; Engel and Moran, 2013; Xia et al., 2013). In addition, the omnivorous nature of *A.suturalis* increases the difficulty of pest control. Previous studies have reported that some symbiotic bacteria help insect hosts form new feeding habits, thus expanding food sources and enhancing the adaptability of insects to the environment (Lu et al., 2001; Douglas, 2009), which may also increase the difficulty of *A.suturalis* control. There are currently few studies examining the symbiotic bacteria of *A. suturalis*, making the number and species of the symbiotic bacteria in *A. suturalis* uncertain (Luo et al., 2021; Ma et al., 2021). Therefore, it is necessary to investigate the distribution of symbiotic bacteria at different stages of *A. suturalis*, which will provide a framework for exploring the function of symbiotic bacteria and pest control in *A. suturalis*.

In this study, the bacterial community composition and relative abundance of 1st instar to 5th instar *A. suturalis* nymphs and 1, 6, and 9 days male and female adults of *A. suturalis* were investigated *via* high-throughput Illumina sequencing of the 16S rRNA gene. To identify new pest control strategies, we explored the cooperative coevolution of *A. suturalis* and its symbiotic bacteria to better understand how *A. suturalis* relates to the symbiotic bacteria community structure, to examine the symbiotic bacteria related to *A. suturalis*, and to provide a theoretical basis for revealing a series of principles such as its resistance regulation mechanism and feeding characteristics.

## Materials and Methods

### Insect Rearing and Maintenance

The *A. suturalis* used in this study were collected from the field in Wuhan (Hubei Province, China). *Adelphocoris suturalis* strains were maintained in climate chambers (75 ± 5% relative humidity, 26 ± 2°C temperature and a 16:8 h, light:dark cycle) and fed green beans and a 5% sugar solution (Lu et al., 2008). *Adelphocoris suturalis* started feeding cotton aphid from the third instar nymph. Cotton aphids are reared on non-transgenic cotton seedlings, living in the same environment as *A. suturalis*.

### Sampling and DNA Extraction

DNA was extracted from whole insects (1st instar nymph, 2nd instar nymph, 3rd instar nymph, 4th instar nymph, 5th instar nymph, and 1, 6, and 9 days male and female adults) using MagPure Stool DNA KF kit B (Magen, China) according to the manufacturer’s instructions. Six biological replicates were set for samples at each developmental stage (females and males were counted as two treatments). Insects were rinsed three times in distilled sterile water prior to DNA extraction (without soaking in ethanol). DNA was quantified using a Qubit Fluorometer with a Qubit dsDNA BR Assay kit (Invitrogen, United States) and the quality was checked by performing an aliquot on 1% agarose gel.

### Library Construction

The variable regions V3–V4 of the bacterial 16S rRNA gene were amplified with the degenerate PCR primers 341F (5'-ACTCCTACGGGAGGCAGCAG-3') and 806R (5'-GGACTACHVGGGTWTCTAAT-3'). Both forward and reverse primers were tagged with Illumina adapter, pad, and linker sequences. PCR enrichment was performed in a 50 μl reaction containing a 30 ng template, a fusion PCR primer, and a PCR master mix. PCR cycling conditions were as follows: 94°C for 3 min, 30 cycles of 94°C for 30 s, 56°C for 45 s, 72°C for 45 s, and a final extension at 72°C for 10 min. The PCR products were purified with AmpureXP beads and eluted in an Elution buffer. Libraries were qualified by the Agilent 2100 bioanalyzer (Agilent, United States). The validated libraries were used for sequencing on an Illumina HiSeq platform (BGI, Shenzhen, China) according to standard Illumina procedures, and generated 2 × 300 bp paired-end reads. The sequences obtained in this study were deposited in the GenBank short-read archive (SRA), accession number PRJNA662509.

### Sequencing and Bioinformatics Analysis

The data obtained from independent sequencing were analyzed separately. Samples were marked as follows: ZL1: 1st instar nymph; ZL2: 2nd instar nymph; ZL3: 3rd instar nymph; ZL4: 4th instar nymph; ZL5: 5th instar nymph; ZM1D: adult male eclosion at 1 day; ZF1D: adult female eclosion at 1 day; ZM6D: adult male eclosion at 6 days; ZF6D: adult female eclosion at 6 days; ZM9D: adult male eclosion at 9 days; and ZF9D: adult female eclosion at 9 days.

Raw reads were filtered to remove adaptors and low-quality and ambiguous bases, and paired-end reads were added to tags using the Fast Length Adjustment of Short reads program (FLASH, v1.2.11; Magoc and Salzberg, 2011) to obtain the tags. The tags were clustered into operational taxonomic units (OTUs) with a cutoff value of 97% using UPARSE software (v7.0.1090; Edgar, 2013) and we used UCHIME (v4.2.40) and Gold database for chimera sequence alignment and detection (Edgar et al., 2011). OTU representative sequences were then taxonomically classified using the Ribosomal Database Project (RDP) Classifier v.2.2 with a minimum confidence threshold of 0.6, and trained on the Greengenes database v201305 by QIIME v1.8.0 (Caporaso et al., 2010). The USEARCH_global (Edgar, 2010) was used to compare all tags to obtain an OTU statistical abundance table for each sample. Alpha and beta diversity were estimated by MOTHUR (v1.31.2; Schloss et al., 2009) and QIIME (v1.8.0; Caporaso et al., 2010), respectively, at the OTU level. Principal component analysis (PCA) in OTUs was plotted with the R package “ade4.” The difference in alpha diversity among groups was compared using Kruskal-Test, with values of *p* ≤ 0.05 considered statistically significant (^*^, 0.01 6< *p* ≤ 0.05; ^**^, 0.001 < *p* ≤ 0.01; ^***^, *p* ≤ 0.001). We used PICRUSt to obtain the KO corresponding to the OTU through the greengene ID corresponding to each OTU, and calculated the abundance of the KO from the sum of the abundances of the OTU corresponding to the KO. We calculated the abundance of each functional category based on the information in the KEGG database and the OTU abundance information. In addition, PICRUSt was used to obtain the levels of metabolic pathway information, and the abundance of each level was obtained.

### Phylogenetic Analysis of the *Erwinia* and *Acinetobacter*

In order to explore the phylogenetic relationship of the two most abundant bacterial genera, we compared the *Erwinia* and *Acinetobacter* DNA sequences obtained by high-throughput sequencing in the NCBI nucleotide (nr) database. Six 16S rRNA fragments belonging to *Erwinia* and 16 16S rRNA fragments belonging to *Acinetobacter* were downloaded from GenBank to construct a phylogenetic tree. The phylogenetic tree analysis of 249 base pairs was carried out. Using MEGA7.0, the phylogenetic tree was constructed by Neighbor-joining method (1,000 bootstraps).

## Results

### General Description of 16S rRNA Gene Sequencing Results

The bacteria of *A. suturalis* were analyzed by Illumina HiSeq *via* the sequencing of the 16S rRNA gene. We obtained a total of 800,836 raw reads and 707,474 clean reads, with an average length of 296 bp. Based on 97% species similarity, we clustered the spliced tags into OTU. The number of OTUs at each developmental stage is detailed in Table 1. We constructed dilution curves for Ace, Chao1, Shannon, Simpson, Good’s Coverage and Observed species, which demonstrated the quality and credibility of sequencing quantity (Supplementary Figure 1). Good’s coverage of all samples was above 99%, indicating that our sequencing results were sufficient to fully estimate the diversity of *A. suturalis* bacterial community (Table 1).

**Table 1:** 16S rRNA gene sequencing data.

Sample	Number of reads	Mean length	Number of OTUs	Chao1	ACE	Shannon	Simpson	Good’s coverage
ZL1	64,316	296.50	589	200.42	204.28	2.46	0.20	0.99
ZL2	64,317	294.83	483	163.68	168.73	1.99	0.24	0.99
ZL3	64,288	296.33	162	61.75	66.73	0.90	0.60	0.99
ZL4	64,325	296.67	147	60.47	63.19	1.02	0.53	0.99
ZL5	64,338	296.83	151	61.28	68.76	1.04	0.52	0.99
ZM1D	64,291	295.67	103	47.67	48.84	0.82	0.54	0.99
ZF1D	64,299	296.83	98	45.81	47.63	0.80	0.59	0.99
ZM6D	64,286	297.33	127	63.06	69.44	1.21	0.41	0.99
ZF6D	64,313	296.67	130	64.41	66.10	1.23	0.39	0.99
ZM9D	64,300	295.67	114	60.38	64.43	1.17	0.41	0.99
ZF9D	64,401	296.67	118	61.75	65.94	1.26	0.38	0.99

ZL1: 1st instar nymph; ZL2: 2st instar nymph; ZL3: 3st instar nymph; ZL4: 4st instar nymph; ZL5: 5st instar nymph; ZM1D: adult male eclosion for 1 day; ZF1D: adult female eclosion for 1 day; ZM6D: adult male eclosion for 6 days; ZF6D: adult female eclosion for 6 days; ZM9D: adult male eclosion for 9 days; and ZF9D: adult female eclosion for 9 days.

### Nymphal Microbiota

**Figure 1:**
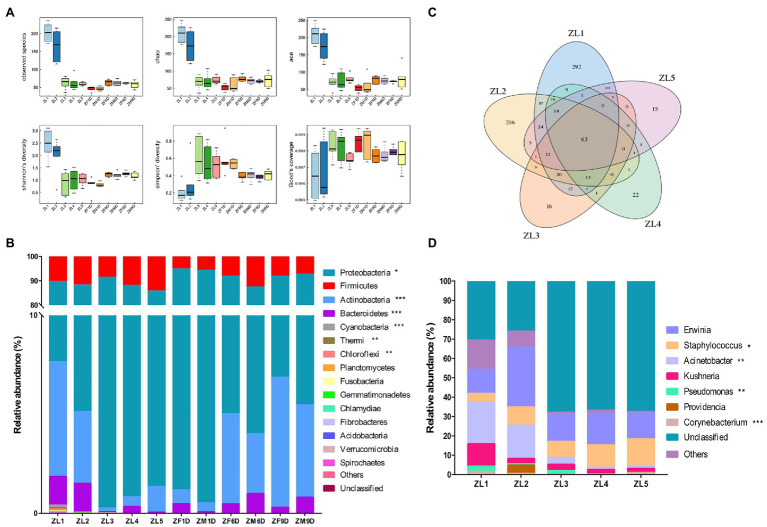
Bacterial community dynamics among different developmental stages in *Adelphocoris suturalis*. **(A)** Boxplot of α-diversity measured by the six indexs. **(B)** Relative abundance of bacteria communities at the phylum level in different groups. **(C)** Venn diagram showing operational taxonomic unit (OTU) classification in nymphal period. **(D)** Relative abundance of bacteria communities at the genus level in nymph stages. (Bacteria with relative abundance lower than 0.5% in all samples were all merged into others. Kruskal-Wallis test, ^*^0.01 < *p* ≤ 0.05, ^**^0.001 < *p* ≤ 0.01, and ^***^p ≤ 0.001).

Nymphs had higher species richness in ZL1 and ZL2 periods, and were significantly higher than other periods (Table 1; Figure 1A). Proteobacteria and Firmicutes were the dominant phyla during the nymphal stage, and their relative abundances were 85.57% (average value across all of the samples at nymphal stage) and 11.34%, respectively (Figure 1B). We compared the changes of *A. suturalis* nymphs in different developmental stages at the genus level. As the nymphs grew, the microbial community changed significantly. There were 63 common OTUs classifications in the five developmental stages of nymphs, and each developmental stage had characteristic OTUs (Figure 1C). *Erwinia* (17.65%) (average value across all of the samples at the nymphal stage), *Staphylococcus* (9.83%), and *Acinetobacter* (9.83%) were the top three genera in relative abundance during the nymphal stages. *Erwinia* was the dominant genus of bacteria throughout the nymph period, and the microbial community was relatively stable without significant dynamic changes. However, the relative abundance of *Staphylococcus, Acinetobacter, Pseudomonas*, and *Corynebacterium* changed significantly (Figure 1D). The relative abundance of *Staphylococcus* increased significantly, the lowest in the ZL1 (4.98%) stage, and the highest in the ZL5 (13.98%) stage. The trends for *Acinetobacter, Pseudomonas*, and *Corynebacterium* were opposite to those of *Staphylococcus*. *Acinetobacter* had the highest abundance in the ZL1 (24.69%) stage, and then gradually decreased (Figure 1D).

### Adult Microbiota

**Figure 2:**
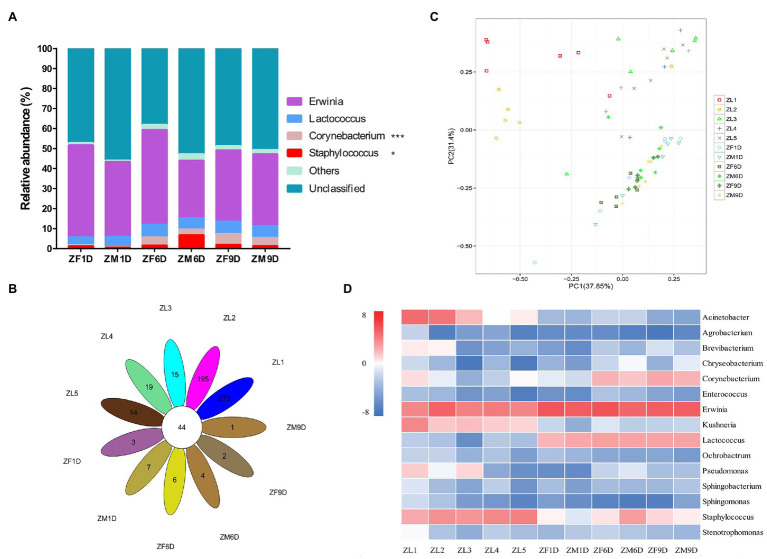
Bacterial community dynamics among different developmental stages in *A. suturalis*. **(A)** Relative abundance of bacteria communities at the genus level in adult stages. **(B)** Core-Pan OTU presents the common and unique OTU of all samples in petal diagram. **(C)** Difference of OTU types in different developmental stages based on Principal Components Analysis (PCA). **(D)** Heat map analysis of the top 15 microbial populations with relative abundance at different developmental stages. The data represented by color in the figure is represented by log2(relative abundance). Bacteria with relative abundance lower than 0.5% in all samples were all merged into others (Kruskal-Wallis test, ^*^0.01 < *p* ≤ 0.05 and ^***^p ≤ 0.001).

As in the nymph stage, the Proteobacteria (88.31%; average value across all of the samples at adult stage) and Firmicutes (7.84%) were the dominant phyla in the adult stage, and the species richness of each developmental stage was similar (Figure 1B). Similarly, we analyzed the bacterial communities of *A. suturalis* adults at different developmental stages at the genus level. *Erwinia* (38.42%; average value across all of the samples at adult stage) and *Lactococcus* (5.53%) were the dominant genus in the entire adult stage and their relative abundance was relatively stable, without significant dynamic changes (Figure 2A). Interestingly, although the relative abundance of *Corynebacterium* was low, it was significantly increased, reaching the highest in ZF9D (5.41%) during the adult stage. Compared with females and males, only *Staphylococcus* had a significant difference on the 6th day of adult development. The relative abundance of *Staphylococcus* ZM6D (6.90%) was significantly higher than that of ZF6D (1.55%), and there was no significant difference in other periods (Figure 2A).

### Comparisons of Bacterial Communities From Different Life Stages

Species richness of ZL1 and ZL2 was the highest (Figure 1A), with 44 common OTU species in all samples, and the unique OTU species of ZL1 and ZL2 was also the highest (Figure 2B). PCA analysis demonstrated that the OTUs of *A. suturalis* in different development stages showed dispersion and aggregation. The degree of dispersioncan show whether the sample composition under the same conditions is similar (Figure 2C). Each point on the PCA graph represented a stage of development; the closer the distance, the more similar the composition.

At the phylum level, taxonomic analysis of all samples showed that Proteobacteria was the most prevalent phylum. Among all samples, the top three phyla with the highest relative abundance were Proteobacteria, Firmicutes, and Actinobacteria, accounting for 87.06, 9.43, and 2.8% (average value across all of the samples), respectively. Proteobacteria had the highest abundance in adult stage compared with nymph stage, with the highest relative abundance in ZM1D (93.77%) stage of adult stage (Figure 1B). Throughout the different development stages of *A. suturalis*, the relative abundance of Proteobacteria increased significantly in the nymph stage, and in ZL3 (91.15%) the relative abundance of the period was the highest. After the first day of adult emergence, the relative abundance of the Proteobacteria showed a decreasing trend (Figure 1B). Compared with Proteobacteria, the change trend of Actinobacteria was the opposite. The relative abundance of Actinobacteria decreased significantly in the nymph stage, and then increased significantly from the first day of adult emergence. The relative abundance of Proteobacteria and Actinobacteria at 6 days (Proteobacteria, ZF6D: 86.86%, ZM6D: 83.39%; Actinobacteria, ZF6D: 4.55%, and ZM6D: 3.01%) and 9 days (Proteobacteria, ZF9D: 84.92%, ZM9D: 87.17%; Actinobacteria, ZF9D: 6.57%, and ZM9D: 4.67%) of adult stage was similar to that at nymph ZL1 (Proteobacteria: 81.97%; Actinobacteria: 5.80%) stage. Firmicutes were also ubiquitous in each developmental stage, and their relative abundance was relatively stable without significant change.

We selected the bacterial genera with the top 15 abundance ratios and drew heat maps based on their relative abundance at different developmental stages (Figure 2D; Supplementary Table 1). From the overall distribution of the microbial community at different developmental stages of *A. suturalis*, *Erwinia* (28.98%; average value across all of the samples) was still the dominant genus (Figure 2D; Supplementary Table 1). *Acinetobacter*, *Kushneria*, and *Staphylococcus* were relatively abundant in the nymph stage, and the number was very small in the adult stage. The abundance ratio of *Lactococcus* during the adult stage was significantly higher than that of the nymph, and the relative abundance during the nymph stage was all below 0.3% (Figure 2D).

### Function Prediction and Phylogenetic Relationship Analysis

**Figure 3:**
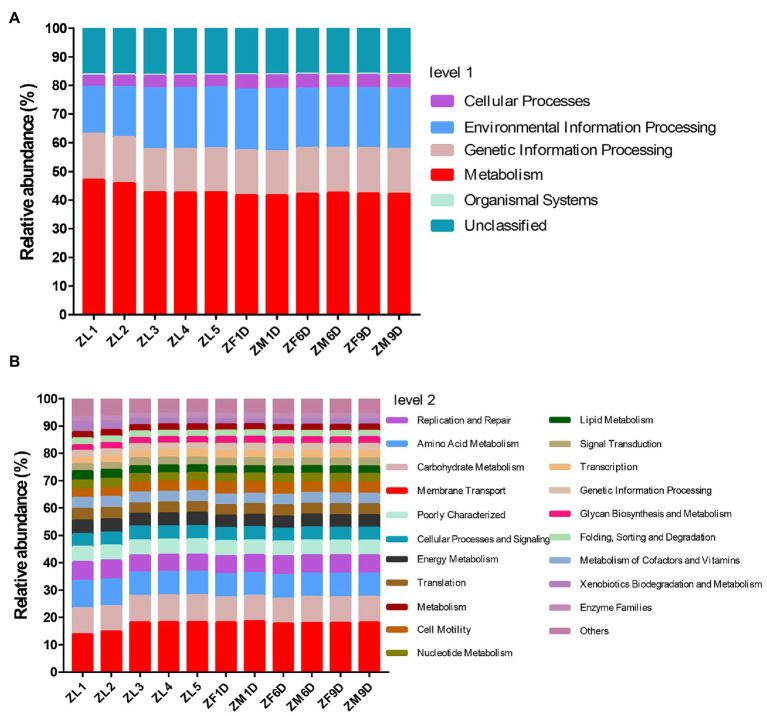
Prediction of functional pathway abundance at different developmental stages. **(A)** Function prediction based on Level 1. **(B)** Function prediction based on Level 2. In level 2, pathways ranked below 20 in total relative abundance were classified as “Others.”

**Figure 4:**
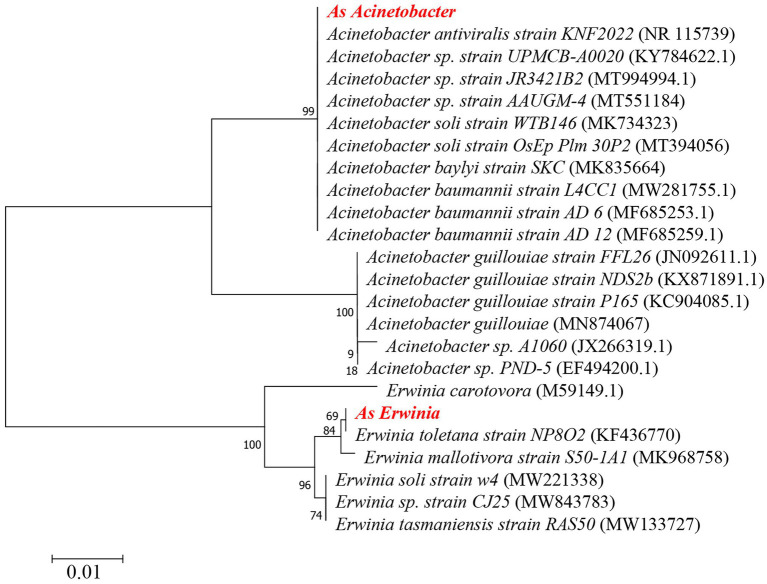
Phylogenetic analysis of *Erwinia* and *Acinetobacter*. *As Erwinia*: *A. suturalis Erwinia*, *As Acinetobacter*: *A. suturalis Acinetobacter*. The phylogenetic tree was made by MEGA7.0 software and constructed by the Neighbor-joining method. The number in parentheses indicates the GeneBank accession number of the 16S rRNA gene sequence.

Based on the predicted results of KEGG function, we showed the pathway abundance at two levels (level 1 and level 2). In level 1, metabolism accounted for about 41.61–47.01% at each developmental stage, followed by environmental information processing (16.46–21.61%) and genetic information processing (15.02–16.02%; Figure 3A). In level 2, we showed the richness of the top 20 pathways, and the other pathways are classified as Others (Figure 3B). The relative abundance of membrane transport (13.89–18.64%) was the highest at different developmental stages. In addition, pathways related to metabolism account for the vast majority, and carbohydrate metabolism, amino acid metabolism, energy metabolism, metabolism of cofactors and vitamins, nucleotide metabolism, lipid metabolism were abundantly enriched. The phylogenetic tree indicated the developmental relationship between *Erwinia* and *Acinetobacter*, the two important genera in our study, and the more closely related genera (Figure 4).

## Discussion

Most insects contain a large number of symbiotic bacteria (Bosch and McFall-Ngai, 2011; Herzner et al., 2013; Ruffner et al., 2013; Marchesi et al., 2016). Although the microbial diversity of insects has been extensively studied, most of the research has focused on insect gut microbes (Roh et al., 2008; Hulcr et al., 2012; Salem et al., 2013), and there are few reports on the dynamic changes of microbial diversity and species richness in different developmental stages of insects. However, the vast majority of insect symbiotic bacteria are concentrated in the intestinal tract, which is rich in symbiotic bacterial communities (Gupta and Nair, 2020). Similarly, although our study detected the microbial community of the entire insect body, intestinal microbes accounted for almost all of them (Campbell et al., 2018; Karimi et al., 2019; Rossitto De Marchi and Smith, 2020). These bacteria and host insects have formed an interdependent relationship during the co-evolution process (Gao et al., 2019). However, we have not found any endosymbionts in *A. suturalis*, although endosymbionts have been widely reported in other Hemipterans (Campbell et al., 2018; Karimi et al., 2019; Rossitto De Marchi and Smith, 2020).

Interestingly, it can be observed from the aspect of microbial diversity that the first and second instar microbial community diversity of newly hatched nymphs is the highest, and its unique OTU species are also abundant. From the third instar nymph, the diversity of the microbial community began to decline. It is well known that bacterial diversities vary from field collected to lab-reared (Rani et al., 2009) as well as across different geographical regions (Zouache et al., 2011). In addition, the composition and diversity of bacteria are affected by the *in vitro* environment (Zouache et al., 2011) and artificial feed (Priya et al., 2012). It has been confirmed in *Helicoverpa armigera* and *Lymantria dispar* that food sources have a great influence on the microbial diversity of insects (Broderick et al., 2004; Priya et al., 2012). *Adelphocoris suturalis* began to prey on cotton aphid from the third instar nymph stage, which may be one of the reasons for the changes in microbial diversity, or it may be that the nymphal microbial community becomes highly simplified through the development of the host insect. Starting from the third instar nymph stage, the alpha diversity decreased significantly (Figure 1A), and diet is the best explanation for this effect. This is consistent with the results of recent studies on the influence of *A. suturalis* diet on microbial diversity (Luo et al., 2021). An increase in nutrient richness (such as protein quality) may lead to a decrease in alpha diversity, although refined diets are also associated with increased species richness (Erkosar et al., 2018; Kešnerová et al., 2020). PCA analysis also confirmed the difference of species diversity mentioned above. During the transition period from fifth instar nymph to adult, there was no significant change in alpha diversity, because *A.suturalis* is an incomplete metamorphosis insect. Nymphs and adults have small changes in their living environment, feeding habits, and food sources, which lead to changes in the intestinal bacterial community (Engel and Moran, 2013; Hammer et al., 2017; Luo et al., 2021). Complete metamorphosis involves complex structural changes, which leads to significant changes in microbial diversity from egg to adult (Chen et al., 2016; Hammer et al., 2017; Zhao et al., 2019).

We also found that Proteobacteria and Firmicutes dominated across the entire life cycle, which was similar to findings in other Hemipteran insects (Husseneder et al., 2017; Lim and Ab Majid, 2020), each of which contained a different proportion of microbes depending on the species or sample (Figure 1B). Similar results have been found in other insects (Chandler et al., 2011; Colman et al., 2012; Engel et al., 2012). These phyla are often listed as the most abundant bacterial communities associated with insect taxa (Colman et al., 2012; Thomas et al., 2013; Yun et al., 2014; Kim et al., 2017). Firmicutes and Proteobacteria are key to maintaining the growth and development of insects during the metabolism of secondary metabolites in host plants (Dillon and Charnley, 2002).

*Erwinia* in the phylum Proteobacteria is a genus of dominant bacteria in the nymphal and adult stages, a member of the Gram-negative Enterobacteriaceae family (Basset et al., 2000), and is a type of intestinal bacteria. Throughout the development cycle of *A. suturalis*, the *Erwinia* population was stable and continuous, with high abundance, indicating that *Erwinia* plays a lasting symbiotic role in the growth, development, and survival of *A. suturalis*. *Erwinia* can metabolize most sources of nitrogen, sulfur, and phosphorus (Friedl et al., 2008), and is thus an important microbial species in the intestinal tract of insects. *Adelphocoris suturalis* is a highly omnivorous insect (Luo et al., 2017b). *Erwinia* can enhance the adaptability of insect hosts to their plant hosts by regulating the diet of insect hosts (De Vries et al., 2004), which is critical for omnivorous insects. *Erwinia* have strong metabolic ability, which plays an important role in the digestion of food and body development of *A. suturalis*. Interestingly, *Erwinia* can secrete a variety of cell wall degrading enzymes, causing potato black leg disease, soft rot, fusarium wilt, and other plant diseases (Whitehead et al., 2002; Grenier et al., 2006). *Drosophila melanogaster* has been reported to be an important media for *Erwinia carotovora* (Vieira et al., 2020). The Hemiptera insect *Creontiades signatus* is a vector for the transmission of bacterial pathogenic bacteria *Serratia marcescens* (Bizio; Enterobacteriales: Enterobacteriaceae) that rots cotton seeds and bolls (Glover et al., 2020). Further research is needed to determine whether *A.suturalis* is a vector for the bacteria. *Acinetobacter* has a very high abundance in the nymph stage, but extremely low abundance in the adult stage. The vast majority of *Acinetobacter* bacteria have strong drug resistance (Li et al., 2021). In China, due to the large-scale planting of Bt crops, *A. suturalis* has risen from secondary pests in cotton fields to primary pests. Among them, Cry1, Cry2, and Cry9 toxins have been reported to show high insecticidal activity against lepidopteran pests (Palma et al., 2014; Silva et al., 2015). Once ingested by the susceptible insect larvae, these cry proteins (present in the form of protoxin) are proteolytically processed by midgut proteases to the active toxin that subsequently binds to specific protein receptors of the midgut epithelium leading to cell disruption and eventual death of the insect larvae (Pardo-López et al., 2013). The introduction of the intestinal isolate *Acinetobacter guillouiae* into *Plutella xylostella* significantly enhances its sensitivity to Bt Cry1Ac protoxin, and *Acinetobacter* plays an important role in the immune response of insects (Li et al., 2021). During the nymph period, its ability to resist the stimulation of external agents is relatively weak. It requires the support of symbiotic bacteria in the body to defend against unfavorable pesticide environments and Bt crops. We analyzed the developmental relationship of *Erwinia* and *Acinetobacter* with the aforementioned *E. carotovora* and *A. guillouiae* through phylogenetic tree development. *As Erwinia* and *Erwinia toletana strain NP8O2* are the most closely related in evolution. At the same time, *As Erwinia* and *E. carotovora* have 94.47% identity. Although *As Acinetobacter* and *A. guillouiae* are not on the same branch, they have 94.86% identity. *Staphylococcus* and *Lactococcus* are the two genera with the highest relative abundance in Firmicutes. The content of *Staphylococcus* increased significantly in the nymph stage, and the relative abundance was the highest in ZL5 (13.98%) stage, but it was scarce in adult stage. *Lactococcus* has a relatively high abundance in the adult stage and exists in large numbers in the intestines (De Jonge et al., 2020). This type of bacteria is known for fermenting complex molecular carbohydrates to produce lactic acid. *Lactococcus* is a lactobacillus of firmicutes with high abundance in both male and female adults, which can decompose sugar to produce organic acids, reduce the pH value of its environment, and defend against some acid-sensitive pathogenic bacteria (Evans and Armstrong, 2006). This ensures that *A. suturali* can obtain the nutrients it needs in the complex environment. Our functional prediction also confirmed that in different stages of development, metabolic function is the main, whether amino acid metabolism or carbohydrate metabolism, is very important for the survival of insects.

Our results showed that the dominant bacteria genera (*Erwinia*, *Acinetobacter*, *Staphylococcus*, and *Lactococcus*) of Proteobacteria and Firmicutes were mostly concentrated in the intestinal tract of insects. These bacteria played an important role in nutrient uptake and adaptability to the environment, and were directly related to the growth, development, and reproduction of the insects. We sequenced the *A. suturalis* 16S rRNA gene through Illumina HiSeq, which directly revealed the structure of the bacterial community in the life cycle of *A. suturali*, predicted the biological functions of different bacterial communities, and provided a basis for further research on the role of bacteria in this and other insects. We provide a crucial theoretical basis for future research on *A. suturalis* symbiotic bacteria. These foundations can help formulate environmentally friendly management strategies for pest control and provide ideas for new pest control strategies.

## Data Availability Statement

The datasets presented in this study cans be found in online repositories. The names of the repository/repositories and accession number(s) can be found at: https://www.ncbi.nlm.nih.gov/genbank/, PRJNA662509.

## Author Contributions

JC and XG: conceptualization and writing – review and editing. XG: methodology. HX, LN, and CW: software. JC: validation and funding acquisition. HX: formal analysis and writing – original draft preparation. HX and XZ: investigation. JL: resources. HX, JJ, and XG: data curation. LW and DL: visualization. KZ: supervision and project administration. All authors contributed to the article and approved the submitted version.

### Conflict of Interest

The authors declare that the research was conducted in the absence of any commercial or financial relationships that could be construed as a potential conflict of interest.

## Supplementary Material

Supplementary Table 1 Relative abundance of bacteria communities at the genus level in different group (Top 15).

Supplementary Figure 1 Alpha diversity dilution curve.
