# Supplementary material for: Gut Bacterial Diversity in Different Life Cycle Stages of Adelphocoris suturalis (Hemiptera: Miridae)
Source: Front Microbiol. 2021 Jun 2;12:670383. doi: 10.3389/fmicb.2021.670383 (PMC8208491; doi:10.3389/fmicb.2021.670383)
Supplement: Supplementary Table 1 — Relative abundance of bacteria communities at the genus level in different group (Top 15). [file Data_Sheet_1.ZIP › Supplementary Material/Supplementary Table 1 Relative abundance of bacteria communities at the phylum level in different group.docx]

Supplementary Table 1 Relative abundance of bacteria communities at the genus level in different group (Top 15)

|  | ZL1 (%) | ZL2 (%) | ZL3 (%) | ZL4 (%) | ZL5 (%) | ZF1D (%) | ZM1D (%) | ZF6D (%) | ZM6D (%) | ZF9D (%) | ZM9D (%) |
| --- | --- | --- | --- | --- | --- | --- | --- | --- | --- | --- | --- |
| Acinetobacter | 24.69 | 18.47 | 3.60 | 1.02 | 1.34 | 0.11 | 0.08 | 0.26 | 0.26 | 0.05 | 0.04 |
| Agrobacterium | 0.25 | 0.01 | 0.03 | 0.04 | 0.01 | 0.02 | 0.01 | 0.01 | 0.01 | 0.01 | 0.01 |
| Brevibacterium | 1.40 | 1.20 | 0.02 | 0.04 | 0.07 | 0.03 | 0.02 | 0.19 | 0.08 | 0.39 | 0.14 |
| Chryseobacterium | 0.32 | 0.09 | 0.01 | 0.09 | 0.01 | 0.08 | 0.03 | 0.29 | 0.85 | 0.07 | 0.58 |
| Corynebacterium | 1.74 | 0.64 | 0.07 | 0.22 | 1.08 | 0.57 | 0.35 | 4.11 | 2.81 | 5.41 | 4.08 |
| Enterococcus | 0.13 | 0.09 | 0.02 | 0.04 | 0.01 | 0.02 | 0.01 | 0.14 | 0.10 | 0.04 | 0.03 |
| Erwinia | 12.07 | 31.90 | 14.43 | 16.55 | 13.27 | 45.90 | 37.29 | 47.36 | 28.61 | 35.58 | 35.76 |
| Kushneria | 12.11 | 2.69 | 3.26 | 2.40 | 2.07 | 0.29 | 0.07 | 0.49 | 0.21 | 0.24 | 0.28 |
| Lactococcus | 0.20 | 0.14 | 0.02 | 0.18 | 0.13 | 3.89 | 5.18 | 6.29 | 5.67 | 6.15 | 5.99 |
| Ochrobactrum | 0.25 | 0.25 | 0.07 | 0.14 | 0.03 | 0.13 | 0.08 | 0.05 | 0.04 | 0.05 | 0.03 |
| Pseudomonas | 2.40 | 0.77 | 1.98 | 0.05 | 0.02 | 0.02 | 0.01 | 0.26 | 0.50 | 0.13 | 0.14 |
| Sphingobacterium | 0.48 | 0.11 | 0.04 | 0.21 | 0.02 | 0.12 | 0.03 | 0.11 | 0.09 | 0.10 | 0.16 |
| Sphingomonas | 0.33 | 0.14 | 0.03 | 0.02 | 0.01 | 0.03 | 0.02 | 0.01 | 0.01 | 0.01 | 0.04 |
| Staphylococcus | 4.98 | 9.86 | 8.59 | 11.75 | 13.98 | 1.19 | 0.51 | 1.55 | 6.90 | 1.96 | 1.34 |
| Stenotrophomonas | 0.87 | 0.15 | 0.06 | 0.20 | 0.12 | 0.09 | 0.17 | 0.26 | 0.16 | 0.10 | 0.09 |

ZL1: 1st instar nymphy; ZL2: 2st instar nymphy; ZL3: 3st instar nymphy; ZL4: 4st instar nymphy; ZL5: 5st instar nymphy; ZM1D: adult male eclosion for 1 day; ZF1D: adult female eclosion for 1 day; ZM6D: adult male eclosion for 6 day; ZF6D: adult female eclosion for 6 day; ZM9D: adult male eclosion for 9 day; ZF9D: adult female eclosion for 9 day.
