# Supplementary figures and images for: Gut Bacterial Diversity in Different Life Cycle Stages of Adelphocoris suturalis (Hemiptera: Miridae)
Source: Front Microbiol. 2021 Jun 2;12:670383. doi: 10.3389/fmicb.2021.670383 (PMC8208491; doi:10.3389/fmicb.2021.670383)

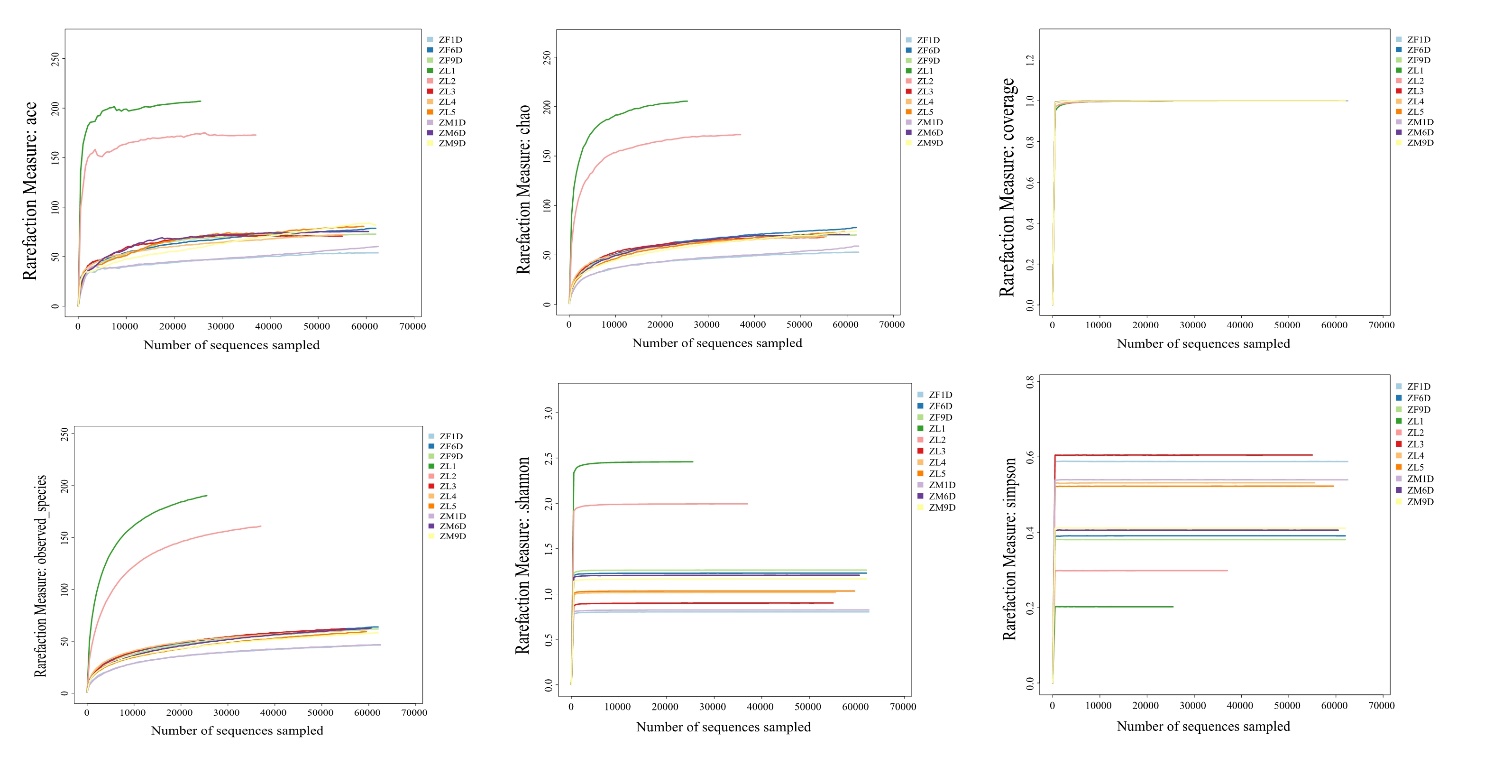


**Supplementary Figure 1.** Alpha diversity dilution curve.

Supplement: Supplementary Table 1 — Relative abundance of bacteria communities at the genus level in different group (Top 15). [file Data_Sheet_1.ZIP › Supplementary Material/Supplementary Figure 1 Alpha diversity dilution curve.docx]
